# Editorial: Brain stimulation and interfacing

**DOI:** 10.3389/fnhum.2023.1179166

**Published:** 2023-04-12

**Authors:** Kai Yu, Junfeng Sun, Bin He

**Affiliations:** ^1^Department of Biomedical Engineering, Carnegie Mellon University, Pittsburgh, PA, United States; ^2^School of Biomedical Engineering, Shanghai Jiao Tong University, Shanghai, China

**Keywords:** brain stimulation, neuromodulation, brain machine interface (BMI), brain computer interface (BCI), human neuroscience, clinical translation, healthcare and well-being

The brain stimulation technologies for human applications, via invasive or non-invasive approaches, provide a means to interact and interface with the brain by injecting electrical current, magnetic field, acoustic waves, or other energy forms (He et al., 2020). These technologies can effectively facilitate certain brain states, augment brain endogenous signals, enable perturbation-based neuroimaging in probing brain connectivity, suppressing specific brain disorders and restoring brain function, etc. The field of human brain stimulation has made tremendous progress in recent years, with new approaches being developed and refined to better understand and modulate brain activity. From the use of electrical/magnetic/acoustic/optical stimulation to treat neurological and psychiatric disorders to the development of brain-machine interfaces to restore sensory, motor and visual functions, the potential applications of brain stimulation are wide-ranging and hold the promise to improve human healthcare and well-being.

The Research Topic of brain stimulation and interfacing is relevant and in concert with the increasing understanding of the underlying mechanisms of brain activities. Advances in neuroimaging and brain stimulation techniques have led to a deeper understanding of the neural circuits and pathways involved in various sensory, motor, visual and cognitive functions. Such increased knowledge allows researchers to investigate brain activities through interfacing with a neuromodulation device. In addition, there is also growing interest in developing brain stimulation and interfacing technologies that enhance memory and attention and gain therapeutic effects for neurological and psychiatric disorders. Brain stimulation techniques, such as transcranial magnetic stimulation, transcranial current stimulation and transcranial focused ultrasound neuromodulation, have been demonstrated to be therapeutic tools for a range of disorders, including depression, chronic pain, Parkinson's disease, and more.

In this Research Topic for the Brain-Computer Interface section of the *Frontiers in Human Neuroscience*, five original research articles address various aspects of non-invasive brain stimulation and interfacing, spanning from transcranial current stimulation to transcranial magnetic stimulation, from computer simulations to clinical trials, from tuning stimulation parameters to determining brain targets. Through these research works with non-invasive brain stimulation modalities, the readers have the opportunity to further understand the significant impact on brain stimulation and interfacing due to individual differences in brain anatomy, specific brain states and targets, types of stimulation approach, and the design of cognitive tasks/paradigms. These are all beneficial explorations toward optimizing the brain stimulation efficiency and thus improving the interface to the brain.

Kashyap et al. did extensive computer simulations on 300 age-sex matched adults and found that the cerebrospinal fluid (CSF) volume of specific brain regions plays an important role and impact the focality of transcranial direct current stimulation (tDCS). Specifically, CSF in the ventricles, which are fluid-filled spaces in the center of the brain, reduces the focality of tDCS, while CSF in the subarachnoid space, which surrounds the brain, increases the focality of tDCS. This suggests that CSF distribution is an important factor to consider when designing tDCS protocols and interpreting its effects. The findings could inform the development of more targeted and effective tDCS interventions for various neurological and psychiatric conditions. However, the study is based on computational modeling and further empirical studies are needed to confirm these simulation results. Serrano et al. implemented a randomized, double-blind, sham-controlled clinical trial and showed that daily treatment at a specific brain region with a home-based tDCS device for multiple weeks ameliorated the cognitive dysfunction due to fibromyalgia. Specifically, active tDCS group showed significant improvement in cognitive performance, particularly in working memory and executive function, compared to the sham group. These findings suggest that home-based tDCS treatment may be a viable option for improving cognitive performance in individuals with fibromyalgia. However, the sample size in this study was relatively small, and the study was conducted over a short period of time. Additionally, the study only assessed cognitive performance and did not investigate other potential benefits of tDCS, such as pain reduction. Further investigation is warranted to identify the optimal treatment parameters for this population. Another interesting work by Peña et al. was to compare the effects of the tDCS vs. the transcranial random noise stimulation (tRNS) in divergent and convergent thinking of more than 60 healthy human subjects. The results of the study showed that both tDCS and tRNS improved divergent thinking, which is the ability to generate multiple solutions to a problem. Moreover, tDCS had a greater effect than tRNS. Additionally, neither type of stimulation had a significant effect on convergent thinking, which is the ability to find the single best solution to a problem. This study provides valuable insights into the effects of different types of non-invasive brain stimulation on divergent and convergent thinking. However, whether the findings in this study can be generalized to the senior adults or patients with neurological or psychiatric disorders needs further investigations.

While the aforementioned works studied the tDCS, Wang et al. investigated the current intensity of occipital transcranial alternating current stimulation (tACS) and correlated the optimal current intensity for two resting-state conditions through EEG recordings. Their results indicate that tACS at different current intensities can modulate resting-state EEG activities in both eyes-open and eyes-closed conditions, with some differences observed between the two conditions. Specifically, tACS at higher current intensities resulted in greater changes in EEG activities, particularly in the alpha band. Moreover, the effects of tACS were more pronounced in the eyes-closed condition than in the eyes-open condition. The authors also suggest potential applications of tACS in clinical and research settings, and highlight the need for further investigation of the optimal parameters for tACS applications. Qu et al. did a systematic review and meta-analysis on 37 published studies covering ~800 human subjects and found that transcranial magnetic stimulation (TMS) leads to a significant neuromodulatory effect on language performance, particularly in tasks related to naming, and semantic/syntactic processing. Specifically, they observed that TMS effects are dependent on several factors, including the stimulation site and parameters, the specific language task, and individual differences in cognitive and neural processing. This work makes an important contribution to the literature on the TMS effects on language performance and provides valuable insights into the potential clinical applications for language disorders. The authors called for future comprehensive studies.

We believe the collected research works are merely several fine examples. Collectively, the *Brain stimulation and interfacing* topic is an area of research that holds great promise for improving human health and well-being, which deserves continuous societal attention and discussion. The articles collected in this topic so far represent the latest developments in this field and provide readers with comprehensive views of the current state of knowledge and the latest advances in this research area. We hope this special topic may be a resource for those interested in this research area. As researchers are continuing to refine and improve existing brain stimulation modalities and brain interfacing technologies, we hope that our collected articles will inspire novel ideas and lead to developments of brain stimulation and brain-machine interface, and that those works will contribute to a deeper understanding of this fascinating and fast-evolving research area.

## Author contributions

All authors listed have made a substantial, direct, and intellectual contribution to the work and approved it for publication.
